# Selective Genicular Artery Embolization in the Management of Osteoarthritic Knee Pain—A Narrative Review

**DOI:** 10.3390/jcm13113256

**Published:** 2024-05-31

**Authors:** Stephanie O’Brien, William G. Blakeney, Julian Soares

**Affiliations:** 1 Department of Orthopaedic Surgery, Royal Perth Hospital, Perth, WA 6000, Australia; 2 Department of Radiology, Royal Perth Hospital, Perth, WA 6000, Australia

**Keywords:** osteoarthritis, embolization, pain, genicular, arthroplasty, arterial, vascular, orthopaedic, arthritis, knee replacement, analgesia, embolic, haemarthrosis

## Abstract

Many people with pain from osteoarthritis (OA) of the knee are either not ready for surgery or may never be surgical candidates. Genicular artery embolization (GAE) is a new proposed management for those with pain despite maximum medical management. It has historically been used to manage recurrent spontaneous haemarthrosis following total knee replacement, but newer studies are showing a positive effect in managing pre-arthroplasty knee OA. The goal of this review is to summarise current and relevant literature from searches of computerised databases and relevant journals, and analyse their results. Studies included show that GAE has promising outcomes in managing mild to moderate OA knee pain in those who have exhausted at least 3 months of conservative therapy. Most studies show improvements in VAS pain and PROM scores (including KOOS, and/or WOMAC). Minimal adverse effects have been associated in up to two years of follow up, the majority of which are self-resolving. The article précises a concise general procedural technique for performing GAE, as well as comparing and contrasting different embolic agents that may be utilised. GAE shows promising outcomes in management of mild to moderate OA knee pain. In the future, there will need to be higher volume studies to determine effectiveness, suitable candidates, and other potential adverse effects.

## 1. Introduction

Osteoarthritis (OA) of the knee is a prevalent disease affecting millions worldwide, with a lifetime symptomatic risk of 40% to 47% [1]. With an ever-ageing population, we can expect the incidence to continue to rise in the coming years. Symptomatic knee OA is managed in a step-ladder technique, starting with physiotherapy, muscle strengthening and weight loss, progressing onto pharmacological therapy in the forms of NSAIDs and intra-articular injections (glucocorticoid and hyaluronic acid), with the final step being arthroplasty [2]. For many years, there has been no good intermediary management for those who have pain despite maximum conservative therapy. This poses a problem for those who do not have severe enough disease to warrant arthroplasty, or for those who are poor surgical candidates.

OA and its associated pain were previously believed to be a degenerative wear-and-tear process, but it is now evident that there is a significant associated inflammatory reaction. Chronic inflammation stimulates angiogenesis, increases the release of inflammatory mediators, causes synovial hypertrophy, increases sensory nerve growth, and subsequently increases pain, advancing bone and cartilage destruction [3]. Progressive joint damage stimulates proinflammatory cytokines which encourage angiogenesis, furthering degenerative chondral wear [4].

Geniculate artery embolization (GAE) is a technique that was first introduced into routine practice for management of spontaneous recurrent hemarthrosis following total knee arthroplasty (TKA) [5]. The first reported documentation of GAE for symptomatic knee OA arises from a 2015 study, which showed a reduction in pain following embolization of the arterial supply to the inflamed synovium [4]. In recent years, there has been a significant increase in novel research that supports the use of GAE as an analgesic benefit. The goal of this review is to summarise the current available data and discuss what is missing from the current literature to help determine which patients will have symptomatic value from GAE in the future.

## 2. Materials and Methods

**Figure 1:**
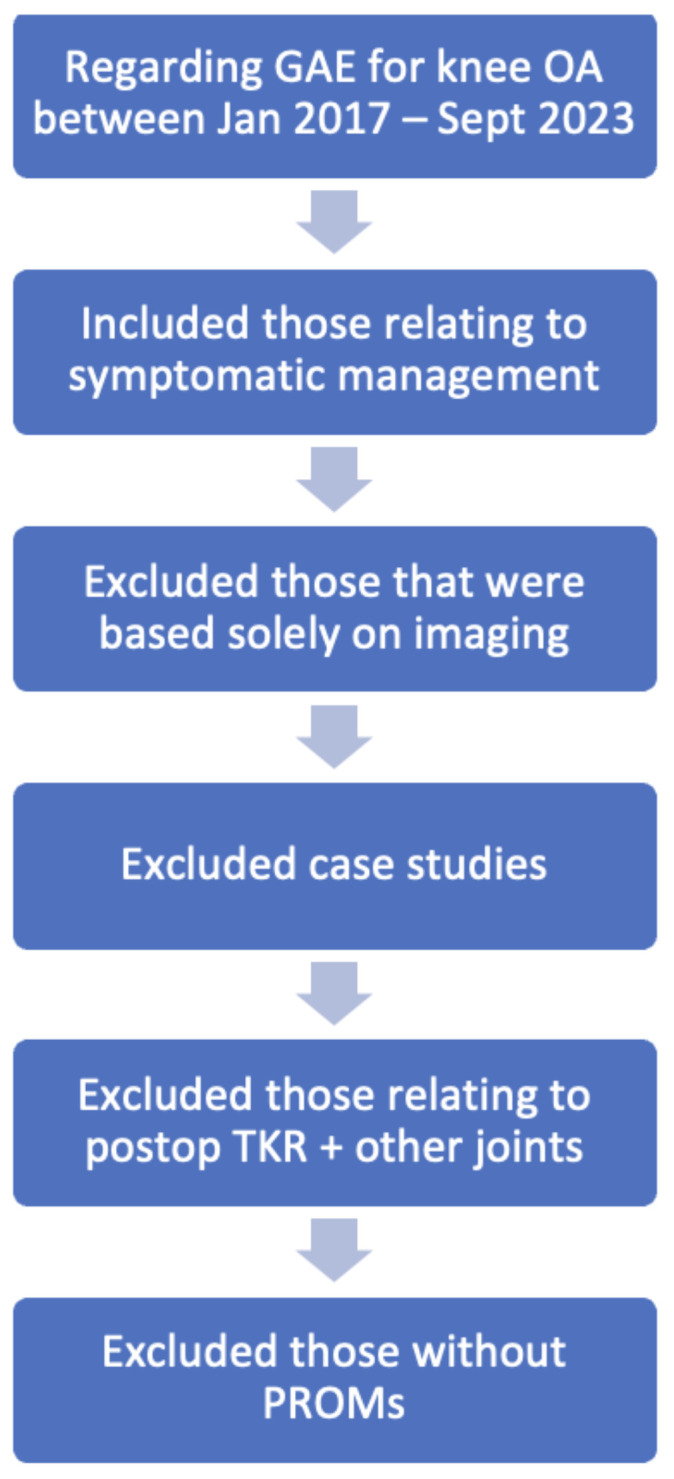
Eligibility Screening of articles.

The information collected for this narrative review has been sourced from the referenced journal articles. The utilised journal articles were retrieved from PubMed searching for keywords: Genicular artery embolization; transarterial embolization. Searched articles ranged from January 2017 to September 2023. Inclusion criteria included recent clinical studies, systematic reviews and meta-analysis, and excluded single case studies. All large-scale studies had their references reviewed, and relevant papers from these were reviewed and included. Articles were excluded when concerned with haemarthrosis post TKA, embolization associated with other joints, or when determining response radiographically rather than symptomatically. Potential articles were reviewed by two independent assessors for consideration of eligibility (Figure 1).

## 3. Results

### 3.1. Patient Factors

Patients deemed suitable for GAE are those who have significant pain due to knee OA that is refractory to conservative treatment. This is considered three to six months of attempted activity reduction, physiotherapy, weight loss, and pharmacological management with no reprieve. Okuno et al. showed good pain relief in those with mild to moderate knee OA [1]. In comparison, Lee et al. examined both mild to moderate as well as severe OA, to determine effectiveness. Severity was determined with a semiquantitative radiographic Kellgren–Lawrence (KL) grading scale and psychometric response and the visual analogue scale (VAS). There was a significant decrease in mean VAS scores at 6 months follow up, which was maintained at 10 ± 3 months post treatment. Although those with severe OA had decreased pain, they overall did not have the same improvement as those with mild–moderate OA [6].

Patients excluded from studies were those with malignancy, significant ipsilateral prior knee surgery, rheumatoid arthritis, irreversible coagulopathy, renal impairment, local infection, and advanced atherosclerosis [5].

### 3.2. Procedural Technique

Those who went on to receive GAE had similar techniques across studies. All were performed by an Interventional Radiologist who was familiar with the genicular arterial anatomy. The goal is to diminish hypervascularity while sustaining arterial flow [5]. Pre-procedure, the clinical localisation of pain was clearly examined and discussed with the patient. Tender areas were marked with radiopaque markers [7]. The most common areas of embolization are branches of the medial and descending genicular arteries, due to the increased burden of disease on the medial compartment [4].

**Figure 2:**
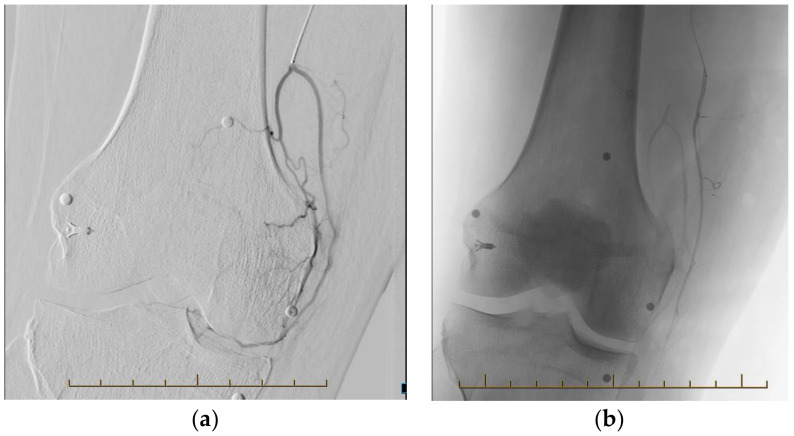
Procedural radiographs before (**a**) and following (**b**) embolization.

A transarterial approach to the genicular arteries is used, most commonly via the ipsilateral femoral artery with antegrade access (alternate studies utilised access via the radial artery) via a 4Fr sheath. An angiogram of the lower limb is performed to define arterial anatomy and collateral blood supply; the latter is to prevent non-target embolization of the osseous component and ligaments of the knee [4]. A 2 French microcatheter is used to select target vessels demonstrating a blush-enhancement on angiography. Any blush which correlated to a site of reported pain was embolised [8]. The chosen embolic agent is injected slowly until the tumour blush ceases and flow is preserved through the target vessel (Figure 2) [7]. Ice packs on the skin overlying the area of embolization produce a cold-induced vasospasm that minimises imprecise arteriole embolization. Injecting nitroglycerine prior diminishes embolization of muscle branches [9]. 

An average of 2–3 vessels were embolised per knee [8]. The patients were able to be discharged the same day 2–4 h post, presuming they had no post-procedure bleeding or complications. In almost all studies, there was a near 100% success rate in intended procedural outcomes.

### 3.3. Embolic Agents

There are now a multitude of embolic agents that can be utilised when performing GAE. The most commonly used is Imipenem/cilastatin (IPM/CS), followed by Embozene microspheres, and less commonly used are resorbable microspheres and polyvinyl alcohol [2].

IMP/CS is more commonly preferred due to its temporary embolic effect. It can be diluted to different particle sizes, with no clear current direction on the optimal particle size. The arterial occlusion lasts up to a few days, with full clinical effect taking a few weeks [3].

The most common alternative agent is Embozene microspheres. These are non-resorbable microspheres that cause permanent vascular occlusion with a rapid onset, showing a more rapid clinical response when compared with temporary embolic agents. This is used in patients with a history of valproic acid allergy, allergy to antibiotics, and hypersensitivity. Comparatively, they have a mildly increased risk of non-target embolization, and increased risk of cutaneous sensory changes and overlying skin discoloration. This risk can be mitigated with the use of ice packs on skin overlying sites of embolization during the procedure [3].

Bhatia et al. completed a sole study comparing use of IMP/CS and Embosphere microspheres in GAE for moderate to severe knee OA with a two year follow up. They concluded an overall decrease in pain, and no significant difference at 2 or 24 months post procedure when comparing Western Ontario and McMaster Universities Arthritis Index (WOMAC) scores [3]. This study is limited by small patient numbers (14 in total who completed the study), was retrospective, and single centre. It does show a promising future for use of Embosphere microspheres with similar outcomes to those treated with IPM/CS, and shows good longevity in symptomatic treatment post procedure.

### 3.4. Grading Function

Assessment of pain and function in most studies is determined with a few key scoring systems. The VAS is a graded scale with increasing numerical value indicating worsening pain. It is used to compare pain progression in a single subject, and is sensitive to changes in improvement [5].

WOMAC and Knee Injury and Osteoarthritis Outcome Score (KOOS) are questionnaires used to evaluate pain during activity, and help quantify stiffness [5].

Rarely, studies may utilise Whole-Organ Magnetic Resonance Imaging Scores (WORMS) to radiographically grade arthritis, although this is less commonly used due to necessity of repeated MRIs during the study process.

## 4. Discussion

There have been many new published studies that show improved symptoms with use of GAE in predominantly mild–moderate knee OA.

Bagla et al. performed a triple-blinded randomised control trial including 21 patients in total. Fourteen underwent GAE, and seven had a sham procedure to treat knee pain secondary to OA. At one month’s follow up, there was a significant improvement of symptoms in the cohort who received GAE when comparing WOMAC and VAS scores. VAS scores showed a reduction in the treatment group at 1 month by 50.1 mm (95% CI, *p* < 0.01). WOMAC scores improved by 24.7 points (95% CO, *p* < 0.02). The improvement was so significant that all those who received the sham procedure went on to have GAE at one month follow up [10]. Limitations include a small centre size, and short follow-up period, with no long-term outcomes for those receiving sham procedure after one month. This was following on from a prior study from Bagla et al. in 2020 assessing the efficacy and safety of GAE in 20 patients. VAS improved from 76 ± 14 at baseline to 29 ± 27 at 6 months following the procedure. WOMAC improved from 61 ± 12 to 29 ± 27 in this time [11]. 

Okuno et al. investigated clinical and MRI changes before and after GAE for a minimum of two years. Pre-procedure MRIs helped guide selective embolization. The 35 included patients also had follow-up MRIs at 2 years following treatment. This confirmed no bony necrosis, no significant cartilage loss, and interestingly a significant improvement on WORMS synovitis (*p* = 0.0016), although overall WORMS score wholly did not significantly change. Clinically, there was significant improvement in pain and symptoms throughout two to four years of follow up (*p* < 0.001). WOMAC scores decreased from 12.1 ± 2.3 at baseline down to 2.6 ± 3.4 at 24 months following GAE, with the largest improvement being seen within one month following the procedure. This is clinically relevant information as, if GAE does improve synovitis, it may help in delaying OA progression [1]. Limitations of this study include small sample size, no control group, and it was an unblinded study. Choi et al. evaluated patients who underwent GAE with an MRI within one month prior to the procedure to determine if MR findings could help determine those who would be responsive to therapy. Twenty-eight knees achieved 3 month follow up without further intervention. They reported no responders in those who had a KL grade 3 or above. Other MRI findings that suggested a patient would be a non-responder included large bone marrow lesions and high-grade meniscal pathology [12]. 

Landers et al. performed a triple-blind RCT comparing GAE vs. sham procedure, and monitored outcomes post procedure. MRIs were performed at baseline and at one year follow-up, which showed no evidence of osteonecrosis. A total of 58 patients (29 in each group) completed the study. Although there was no significant difference in outcomes at one month follow up, a subgroup analysis showed that those patients who underwent complete embolization rather than partial embolization had improvement in KOOS scores, which was also a significant improvement compared to the control group (*p* = 0.012). KOOS scores improved across all five measured parameters, including median KOOS pain (47.2 to 72.2), median KOOS (50 to 78.6), KOOS sports/rec (15.0 to 75.0), KOOS ADL (51.5 to 86.8), and KOOS QOL (18.8 to 62.5). As this was a finding from a subgroup analysis, it requires further studies to confirm if there is a possible dose-dependent effect in GAE [13].

Heller et al. reviewed and compared notable GAE studies to date, and analysed results including timing of clinical effect following GAE using WOMAC, KOOS, and VAS scores. Significant improvement was seen from as early as one day post procedure, with 80–86% having clinical success at the six month follow up. The clinical response lasted between 6 and 24 months [4]. Padia et al. performed a prospective single-centre trial on efficacy of GAE for moderate to severe knee OA in 40 patients. WOMAC total scores improved from a baseline of 52 by 61%, while VAS improved from a baseline of 8 by 67% at 12 month follow up [14]. Sun et al. also performed a prospective study of efficacy of GAE on 23 knees (17 patients), with a 6 month follow up; 95.6% had clinical improvement in WOMAC and VAS scores at one month post procedure, where VAS improved from 6.6 ± 0.9 to 2.8 ± 1.5 at 6 months, while WOMAC improved from 47.0 ± 12.0 to 20.5 ± 11.0 [15].

Russu et al. investigated a small sample size (n = 17) comparing GAE in a Romanian population. Their outcomes showed significant changes in WOMAC scores and increased KOOS at 1 month following GAE, with no further significant improvement thereafter. WOMAC improved from 49.5 ± 13.2 at baseline to 59.8 ± 12.6 at one month, while KOOS scores improved from 46.6 ± 13.2 at baseline to 56.5 ± 13.9 at one month following GAE [9]. Bedros et al. similarly reviewed 16 patients post GAE, and found they had a significant improvement in pain, stiffness, and function following embolization. VAS scores improved from 68.3 ± 11.8 to 31.9 ± 22.5 at one month, and further to 17.7 ± 16.0 at 12 months, which was statistically significant. WOMAC improved from 43.1 ± 13.9 at baseline to 21.3 ± 13.5 at 3 months, and continued to improve up to the 12 month follow up. They recommended use of follow up MRI to determine incidence and clinical implications of osteonecrosis due to GAE [7]. These studies are limited by small size, a short period of follow up (1 year), and, lastly, no control group.

Torkian et al. also completed a systematic review and meta-analysis including 11 studies and 268 knees. They concluded arthritic knee pain following GAE improved even within one week following GAE. They recommended it as a reasonable and safe option to alleviate pain and decrease analgesic use long term, as well as to improve functional status [2]. Another systematic review and meta-analysis by Taslakian et al. was published in 2023. They concluded that those with a greater knee pain severity at baseline had an improved response to GAE, specifically those with a VAS > 50 [16]. A third systematic review and qualitative analysis by Casadaban et al. in 2020 reviewed clinical outcomes following GAE. They noted VAS decreased throughout follow up, from as early as one day to as late as two years post GAE, most effectively for mild–moderate arthritic knees. In comparison, those with severe chronic OA showed improved VAS at one month post, but this was not sustained at the three to six month follow up [8]. A systematic review and meta-analysis conducted by Epelboym et al. also confirmed patients undergoing GAE had consistent improvements from baseline at one, three, six, and twelve months post GAE [17]. Lastly, the most recent study from October 2023 by Poursalehian et al. completed a comprehensive review, which found a consistent decrease in VAS scores observed across all studies, with maximal improvement between 1 and 4 months post GAE [5]. 

There seems to be the importance of synovitis associated with the pain, and it will be important to differentiate the use of GAE in those with osteoarthritic knee pain without significant synovitis [5].

### 4.1. Post Arthroplasty Knee Pain

GAE is also hypothesised to assist in managing persistent knee pain despite arthroplasty. Up to 20% of patients who undergo knee arthroplasty claim to continue to experience pain, of which 10–15% is unexplained [18]. Chau et al. explored the effectiveness of GAE for those experiencing chronic pain post TKR. This was a single-centre, single-arm study including 12 patients, with a mean interval of 2.8 years following TKR, all experiencing pain since surgery. Those with potential alternative underlying causes of pain were excluded [18]. All patients were found to have abnormal synovial hypervascularity. The mean number of arteries embolised following TKR compared with management of knee OA was lower [18]. The majority (58%) had clinical improvement in KOOS scores at 3 to 6 month follow up. VAS scores at rest and walking decreased by 72% and 41%, respectively. In contrast, 30% had a transient increase in pain for two weeks following embolization [18].

These are still preliminary data regarding managing unexplained chronic pain post TKR so should be viewed with caution. There were a few patients for which the pain increased, although, overall, the outcomes showed improved results for up to 12 months [18].

### 4.2. Adverse Effects

Throughout the aforementioned studies, only mild to moderate adverse effects were reported. The most common treatable and predominantly self-resolving complications included puncture site haematoma, transient cutaneous skin changes of the treated knee, associated erythema, mild fever, and plantar sensory paraesthesia (this became less prevalent when embolic particle size was >75 μm) [2,4,6].

Post embolization syndrome is a self-limiting side effect that manifests as knee pain, low-grade fever, or malaise hours to days following GAE [5]. The majority of these resolved spontaneously without intervention. Puncture site haematoma and bleeding were considered a moderate adverse effect when resultantly the patient had to remain in the hospital for extra hours for observation to ensure resolution. 

Major concerns that will need to be monitored in further studies include associated osteonecrosis secondary to reduced vascularization. Multiple studies recommend the use of follow-up MRI to monitor this. It is also prudent to monitor for knee instability and associated weakness [13].

### 4.3. Future for Research

The concept of selective GAE for managing osteoarthritic associated pain is still a new intervention. There are many areas of opportunity available for future research to determine which patients may show symptomatic benefit. In particular, broader and more specific studies determining effect in mild vs. severe arthritic progression would help determine those that may be suitable candidates. Further data will be required to determine if there is an association with improved response and number of arteries embolised. Larger cohort randomised control trials and, furthermore, large-scale systematic reviews will be required to determine long-term response. 

## 5. Conclusions

In summary, GAE has a promising future as an intermediate intervention to help manage knee pain due to OA, particularly in patients who are not adequately managed with conservative treatments, but are either not yet requiring surgery, or are not suitable candidates. Most studies show improvements in pain when comparing VAS, KOOS, and/or WOMAC scores.

The majority of these studies are small-scale, single-centre studies that have been published in the last five years. There are limited data in regard to randomised control trials, and comparison with sham procedures. This will be an important step moving forwards in research to clarify outcomes. Through future studies, we can expect to determine the long-term outcomes, results, and possible adverse effects associated with GAE. The average follow up in most studies was between one and two years post GAE.

## Data Availability

The original contributions presented in the study are included in the article; further inquiries can be directed to the corresponding authors.
